# Dream-reality confusion in borderline personality disorder: a theoretical analysis

**DOI:** 10.3389/fpsyg.2015.01393

**Published:** 2015-09-15

**Authors:** Dagna Skrzypińska, Barbara Szmigielska

**Affiliations:** Unit of Sleep Psychology, Institute of Psychology, Jagiellonian UniversityKrakow, Poland

**Keywords:** dream-reality confusion, borderline personality disorder, sleep disturbances, dissociation, cognitive disturbances, dream content, boundaries

## Abstract

This paper presents an analysis of dream-reality confusion (DRC) in relation to the characteristics of borderline personality disorder (BPD), based on research findings and theoretical considerations. It is hypothesized that people with BPD are more likely to experience DRC compared to people in non-clinical population. Several variables related to this hypothesis were identified through a theoretical analysis of the scientific literature. *Sleep disturbances*: problems with sleep are found in 15–95.5% of people with BPD (Hafizi, 2013), and unstable sleep and wake cycles, which occur in BPD (Fleischer et al., 2012), are linked to DRC. *Dissociation*: nearly two-thirds of people with BPD experience dissociative symptoms (Korzekwa and Pain, 2009) and dissociative symptoms are correlated with a fantasy proneness; both dissociative symptoms and fantasy proneness are related to DRC (Giesbrecht and Merckelbach, 2006). *Negative dream content*: People with BPD have nightmares more often than other people (Semiz et al., 2008); dreams that are more likely to be confused with reality tend to be more realistic and unpleasant, and are reflected in waking behavior (Rassin et al., 2001). *Cognitive disturbances:* Many BPD patients experience various cognitive disturbances, including problems with reality testing (Fiqueierdo, 2006; Mosquera et al., 2011), which can foster DRC. *Thin boundaries*: People with thin boundaries are more prone to DRC than people with thick boundaries, and people with BPD tend to have thin boundaries (Hartmann, 2011). The theoretical analysis on the basis of these findings suggests that people who suffer from BPD may be more susceptible to confusing dream content with actual waking events.

## Introduction

Dream-reality confusion (DRC) is a difficulty or inability to determine whether an event or experience occurred during the waking state or whether it was part of a dream. Although, only few studies on DRC in non-clinical populations have been conducted (e.g., Johnson et al., 1984; Mazzoni and Loftus, 1996; Rassin et al., 2001; Kemp et al., 2003), DRC has been investigated in specific groups, including narcolepsy patients (Wamsley et al., 2014). Research has found that there is a relationship between DRC and psychotic symptoms (e.g., Hempel et al., 2003), but the authors of the present paper have not been able to find any scientific studies on the relationship between DRC and borderline personality disorder (BPD).

BPD is *a pervasive pattern of instability of interpersonal relationships, self-image and affect, and marked impulsivity that begins by early adulthood and is present in a variety of contexts* (DSM-V; American Psychiatric Association, 2013, p. 663). To qualify for this diagnosis, the person should, among other symptoms, make a frantic effort to avoid real or imaginary abandonment, experience a chronic feeling of emptiness or stress-related temporary paranoid symptoms, or exhibit severe dissociative symptoms. Moreover, persons with BPD often engage in self-destructive behaviors and are at significant risk of suicide. Borderline personality disorder affects between 1 and 5.9% of the general population (Torgersen et al., 2001; Aragonés et al., 2011).

Due to the complex psychopathology of BPD, numerous studies have examined different areas of functioning in individuals with this disorder. The present theoretical analysis addresses the question of whether individuals with certain features of BPD may have difficulty distinguishing between dreams and reality.

The aim of this paper is to provide an overview of the current knowledge of DRC in relation to features of BPD. We hypothesize that BPD subjects are highly predisposed to experience DRC. This hypothesis is supported by the underlying assumption that there are groups of interrelated variables that are present in both DRC and BPD. These variables, which we identified through an analysis of the scientific literature, can be divided into the following categories: (i) sleep disturbances; (ii) dissociative symptoms; (iii) negative dream content; (iv) cognitive disturbances; and (v) thin boundaries. This division was made on the basis of theoretical considerations; no factor analyzes have been conducted yet. Each of these five variables is presented separately below.

## Variables Present in BPD and DRC

### Sleep Disturbances

Sleep disturbances, for the purpose of this theoretical analysis, include a variety of problems with sleep that are discussed below. Such sleep problems are very common among individuals with BPD (Hafizi, 2013). Though there is little epidemiological data on sleep disorders among persons diagnosed with BPD, cross-sectional studies show that sleep disorders are prevalent in 15–95.5% of this group (e.g., Asaad et al., 2002; Semiz et al., 2008; Plante et al., 2009; Sansone et al., 2010).

Compared to a non-clinical group, individuals with BPD take more time to fall asleep, sleep for shorter times, have lower sleep efficiency, and have frequent sleep disturbances (Semiz et al., 2008). EEG recordings showed, for example, that study participants in a BPD group, compared to a non-clinical population, had shorter NREM sleep stages 2 and 4 and longer NREM sleep stage 1, and had high-voltage delta waves during NREM sleep (Benson et al., 1990; Philipsen et al., 2005). REM sleep also was different between the groups, with BPD patients spending more time in REM sleep, which had a longer latency, a longer first episode, and a higher REM density, as well as high-voltage delta and theta waves in REM sleep, in participants with BPD (Asaad et al., 2002; Philipsen et al., 2005). Patients with BPD also have more night awakenings than persons from a non-clinical population (Battaglia et al., 1993; La Fuente et al., 2001). Frequent awakenings may lead to difficulty determining whether an event/experience occurred during the waking state or was part of dream content (Trajanovic et al., 2007).

Labile sleep–wake cycles are another example of sleep disturbances. They may occur in the course of BPD and they are correlated with DRC (Fleischer et al., 2012). Labile sleep–wake cycles may promote the intrusion of dreamlike experiences into waking consciousness that can lead to DRC and foster the feeling of depersonalization, which is a dissociative symptom. They also have an adverse effect on memory, thus favoring the creation of false memories (van der Kloet et al., 2012). Individuals who report sleep disturbances score high on dissociative scales, fantasy proneness (a tendency for *deep and long-standing involvement in fantasy and imagination;* Lynn and Rhue, 1988, p. 35) and are prone to false memories (Giesbrecht and Merckelbach, 2006).

Taken together, the above relationships appear to support our hypothesis that BPD patients are likely to experience DRC. It is suggested that a wide range of sleep disturbances, such as labile sleep–wake cycles, increase the tendency to experience night awakenings or nightmares (which are discussed in Section “Negative dream content”) in patients with BPD, which increases the probability that such persons will have problems distinguishing whether an event/experience occurred during the waking state or was part of dream content.

### Dissociative Symptoms

Dissociation describes a state of disruption and/or discontinuity in integrated psychological functioning, such as consciousness, memory, identity, or perception (DSM-V; American Psychiatric Association, 2013). Dissociative symptoms include, derealization – the impression that the surrounding world or reality have changed, depersonalization – feeling as though one is an outside observer of one’s own self, and amnesia – an inability to remember, store, and/or evoke memories.

Dissociative states are experienced by approximately 2/3 of patients with BPD (Korzekwa and Pain, 2009). Persons diagnosed with BPD have a stronger tendency toward dissociative symptoms than non-clinical population and individuals who suffer from depression or schizophrenia (Merckelbach et al., 2005). The occurrence of dissociative symptoms during the course of BPD may be associated with childhood traumatic events. According to one of the theories of the etiology of BPD, this personality disorder develops in individuals who report that traumatic events were a characteristic of their early lives, mainly physical abuse and emotional neglect. A study of 139 patients with BPD found that those who had high scores on the *Dissociative Experience Scale* (DES), which measures the frequency of dissociative symptoms, such as autobiographical amnesia, derealization, depersonalization, absorption, and identity alteration (Bernstein and Putnam, 1986), experienced significantly more severe emotional and physical neglect and emotional and physical abuse (but not sexual abuse) during childhood than those who had low scores on the DES (Watson et al., 2006). The results suggest that individuals exposed to severe traumatic events during childhood are more likely to develop dissociative symptoms. Traumatic experiences may contribute to the development of dissociative symptoms because trauma-related stress leads to a sudden discontinuity of the individual’s experience, as it arouses fear about security and disturbs the connection between the inner and outer environment. Traumatic experiences also often interfere with the integration of mental functions, thus, leading to their dysfunction (Vermetten and Spiegel, 2014). Moreover, dissociative symptoms involve automatic avoidance strategies that defend consciousness from traumatic memories (Briere, 2002).

It is noteworthy that dissociative symptoms are one of the correlates of DRC (Rassin et al., 2001). Levitan (1967, p. 157) noted that *derealization is a compromise between dreaming and waking.* It seems that frequent experiences of dissociative symptoms or their intensification may produce frequent intrusions of dreams into experiences during the waking state.

Dissociative symptoms and proneness to fantasy – characteristics linked to DRC – are correlated, and it appears this correlation can be mediated by experiences during sleep (Giesbrecht and Merckelbach, 2006). High fantasy-prone students report more dissociative symptoms than their friends who score low or medium on fantasy-proneness (Rauschenberger and Lynn, 1995; Waldo and Merritt, 2000). Furthermore, individuals who find it difficult to discriminate between dreams and reality score higher on scales that measure dissociative symptoms and fantasy proneness than individuals who do not confuse dream content with experiences during the waking state (Rassin et al., 2001). A study of 51 women from the general population found that fantasy proneness is linked to both dissociative symptoms and everyday cognitive failures (Merckelbach et al., 1999). Moreover, dissociative symptoms, fantasy proneness, cognitive failures, and sleep disturbances are correlated (van Heugten – van der Kloet et al., 2014a). Later in the current paper, we present data indicating that disturbances in cognitive functioning are among the variables that increase proneness to DRC.

The relationship between dissociative symptoms and fantasy proneness also has been observed in clinical populations. Merckelbach et al. (2005) demonstrated such relationships in groups of subjects diagnosed with BPD, schizophrenia, and major depressive disorder. In addition, Steiger et al. (2000) found that dissociative symptoms were related to impulsivity in persons with BPD. This association is interesting because impulsivity is one of the diagnostic features of BPD (DSM-V, American Psychiatric Association, 2013).

To summarize, the above findings support our hypothesis that individuals with diagnosed BPD are more likely to experience DRC because of their tendency to experience dissociative symptoms and related phenomena, such as fantasy proneness, sleep disturbances, and cognitive problems.

### Negative Dream Content

Individuals suffering from BPD experience more negative life events than other individuals – even those with other personality disorder**s** (Pagano et al., 2004). According to the classical continuity hypothesis (Hall and Nordby, 1972; Schredl, 2003), dreaming reflects the dreamer’s waking life experience, therefore, the dream content of patients with BPD may be more negative that the dream content of other people. The quantitative analysis of a group of 27 individuals diagnosed with BPD and a non-clinical group of 20 individuals showed that the BPD group had dreams with more negative affect than those in the non-clinical group. Generally, individuals suffering from BPD experience negative dreams, including nightmares, more often than individuals who do not have any of the characteristic symptoms of this personality disorder (Schredl et al., 2012).

Nightmares are sleep disturbances that are related to sleep disorders. They are defined as vivid dreams, charged with negative emotions that awaken the dreamer from sleep (DSM-V; American Psychiatric Association, 2013). About 49% of patients with BPD are troubled by nightmares, whereas the prevalence of nightmares in the non-clinical population is estimated to be about 4–10% (Levin and Nielsen, 2007; Simor et al., 2010). The higher frequency of nightmares among BPD patients compared to the non-clinical population is related to greater emotional instability and heightened neuroticism in this clinical group (Simor et al., 2010). The intensity of BPD symptoms is positively correlated with the frequency of nightmares (Semiz et al., 2008). To try to explain the prevalence of nightmares in persons with BPD, we present two theories: a nightmare model proposed by Levin and Nielsen (2007), and the Emotional Cascade Model developed by Selby et al. (2013).

Levin and Nielsen (2007) proposed a theory to explain the occurrence of dysphoric dreaming, which is based on two major assumptions: *cross-state continuity* and *multilevel explanation*. The first, *cross-state continuity*, assumes that *some structures and processes implicated in nightmare production are also engaged during the expression of pathological signs and symptoms such as dissociative symptoms during the waking state* (Levin and Nielsen, 2007, p. 495). The second, the *multilevel explanation*, refers to the idea that nightmare formation can be explained at two different levels: the cognitive–emotional level and the neuronal level. At the cognitive–emotional level, there are imagery processes that represent emotional dream imagery, whereas the neuronal level contains a network of brain regions responsible for imagistic and emotional processes. This model was created to explain the occurrence of nightmares in the course of posttraumatic stress disorder (PTSD); however, it may also be used in an attempt to describe experiences related both to nightmares and cross-state continuity in patients diagnosed with BPD. We will not discuss the concept of neuronal correlates of DRC and BPD, as this is beyond the scope of the present article. Instead, we will focus on the notion of *cross-state continuity* with reference to BPD. In their model, Levin and Nielsen (2007) consider a number of factors that underlie *cross-state continuity*, such as affect load – *a state factor is the combined influence of stressful and emotionally negative events on an individual’s capacity to effectively regulate emotions* (p. 497), and affect distress – *a trait factor is a long-standing, dispositional tendency to experience heightened distress and negative affect, and to react with extreme behavioral expressions* (p. 498). Other factors include high degrees of physiological and psychological reactivity, maladaptive coping, fantasy proneness, imagery vividness, and thin boundaries. Numerous studies suggest that almost all of these factors are usually present during the course of BPD, however more recent studies indicate that there is no heightened physiological reactivity in BPD (e.g., Linehan, 1993; Merckelbach et al., 2005; Hunt, 2007; Kuo and Linehan, 2009; Hartmann, 2011; Mosquera et al., 2011; Cavazzi and Becerra, 2014). Persons diagnosed with this personality disorder are characterized by emotional dysregulation, which is the inability to flexibly respond to and manage emotions, entailing emotional sensitivity, heightened and labile negative affect, a deficit of appropriate regulation strategies, and a surplus of maladaptive regulation strategies (Carpenter and Trull, 2013). In addition, BPD entails affective instability and a low level of emotion recognition (Cole et al., 2009).

According to Linehan’s (1993) biosocial theory, which explains the etiology of BPD, this personality disorder develops in children (i) who have a biological propensity to experience negative affect, and (ii) who are in an invalidating and toxic social environment. In BPD, the biological sensitivity to emotional stimuli, heightened negative affect, and a slow return to emotional baseline are connected with difficulty in discriminating and labeling one’s own emotional states, initiating adequate coping strategies to manage these emotional states, and being able to reduce intense negative affective states. Studies confirm that BPD patients display a negative distortion in the identification of their own emotional states and the emotional states of other persons (e.g., Morey, 2014). The inability to accurately recognize emotional states may intensify negative affect, emotional instability, and emotional reactivity in everyday life. Furthermore, patients with BPD are unable to tolerate distress and they usually use maladaptive regulation strategies to cope with distress and the negative emotions they experience, such as ruminations, impulsive behaviors, or cognitive avoidance (Carpenter and Trull, 2013). Disorders of emotional processes in patients with BPD seem to occur not only in the waking state, but also during dreaming, as in the case of nightmares (Simor et al., 2010). We hypothesize that waking and dreaming states may involve similar experiences; therefore, persons with BPD may have difficulty discriminating whether an event/experience occurred during the waking state or was part of dream content.

Emotional dysregulation, which is one of the factors associated with nightmares in Levin and Nielsen’s (2007) theory, is also mentioned by Selby et al. (2013) in their Emotional Cascade Model (ECM). The ECM attempts to explain the etiology of bad dreams experienced in the course of BPD. The effects of nightmares and other bad dreams, apart from the fear they produce, can involve deficits in appropriate emotion-regulation skills, and decrease ability to cope with distress during the subsequent day, according to the ECM. Patients with BPD experience emotional cascades during the waking state, and this negative affect induces rumination – repetitive thoughts with mainly negative content. Ruminations increase negative affect, which, in turn, intensify ruminations. These processes result in increased cognitive activity during sleep that favors the appearance of nightmares and maladaptive behaviors during the waking state that are intended to cope with negative emotions. It seems that frequent nightmares in persons with BPD may influence the occurrence of negative life events (Selby et al., 2013). Generally, a simplified model of emotional cascades assumes the subsequent occurrence of: negative emotional experiences during wakefulness → increase in ruminations → escalation of negative emotions → increase in ruminations → a very aversive emotional state → / possible dysfunctional coping skills connected with affect regulation (e.g., self-harm, substance use) / → elevated cognitive activity during sleep connected with emotional cascades during the waking state → nightmares that are sleeping emotional cascades → nightmares may increase negative affect and vulnerability to stressors during wakefulness → negative emotional experiences during wakefulness… (for a detailed description, see Selby et al., 2013).

Elevated cognitive arousal during sleep may cause awakenings or semi-awakenings, which consequently may lead to difficulty distinguishing between dreaming and waking experiences (Trajanovic et al., 2007; Selby et al., 2013). In addition, the inability to cope effectively with stressful situations may enhance the tendency toward dissociative states (Mosquera et al., 2011), which finally may result in an increased proneness to DRC.

Moreover, a study by Rassin et al. (2001) demonstrated that dreams that are more likely to be confused with reality are realistic, permeated with negative affect, and give rise to behavior in the waking state. Yet, because BPD patients have more negative experiences in both the waking state and sleeping state and more cognitive disturbances, they may have more difficulty recognizing whether an event/experience occurred during the waking state or was part of a dream. Taken together, the findings suggest that frequent unpleasant dream content in BPD may be a factor that increases proneness to DRC.

### Cognitive Disturbances

Patients with BPD can experience a number of different cognitive disturbances. These include problems with metacognitive monitoring, which refers to the ability to observe one’s own thinking processes, as well to detect errors in one’s own reasoning, and inconsistencies in one’s own narrations. Metacognitive monitoring refers to “thinking about thinking.” Metacognitive knowledge, which is the ability to notice that some things/events may not be as they appear to be, is also distorted in BPD (Sharma and Singh, 2012). Usually, executive functions, such as working memory and response inhibition, also are disturbed in BPD (Hagenhoff et al., 2013). Moreover, BPD is characterized by deficits in feedback processing, altered social inference, and poor decision-making skills (Trivedi, 2006; Mak and Lam, 2013). Generally, four types of cognitive disturbances are distinguished in BPD: (i) transient, quasi-psychotic cognition, (ii) dissociation, (iii) social cognitive biases, and (iv) neurocognition (Fertuck and Stanley, 2006). A detailed description of cognitive problems in BPD, however, remains beyond the scope of the present paper.

What is important is that problems with reality testing may occur in patients with BPD (Fiqueierdo, 2006). Reality monitoring, which is related to reality testing, seems to play a significant role in the process of distinguishing dream content from waking experiences. Reality monitoring, a type of source monitoring, is defined as the ability to discriminate between memories of actual events, and memories of dreamed events, imagination, or delusions. Reality monitoring consists of two decision-making processes: (i) evaluation of whether the characteristics of a memory trace are more typical of memories of external events or memories generated internally; and (ii) evaluation based on present memory/knowledge, which is a more complex process and takes more time. Memory source is distinguished on the basis of its characteristics: memories of actual events include more perceptual and contextual details, whereas memories of consciously imagined events include traces of cognitive operations that were involved in their creation. Dreams are classified as internally generated events, which are difficult to distinguish from similar, external events because they are created without conscious cognitive operations (Johnson et al., 1984). In the case of dreams, conscious cognition, which is the most important cue that would help differentiate between internally generated memories and those generated externally is not present. These conclusions indicate that DRC may be associated with difficulty with reality monitoring.

The temporary suspension of the source monitoring process, along with reduced ability to respond to a sensory stimulus and reduced attention, is one of the common features of both dreaming and waking fantasy. These processes may make it more difficult to distinguish between the content generated during dreaming and waking fantasy. Both waking fantasy and dreams play an integral role in mood regulation, adaptive information processing, and maintenance of self-cohesion by providing working templates for future goal-directed behavior and the development and maintenance of self-schemas (Levin and Young, 2002). BPD patients exhibit certain cognitive disturbances that make them more prone to problems related to reality testing, and waking fantasy may also disturb the processes involved in correct source monitoring. Furthermore, it seems that mood regulation is disturbed in BPD because of more negative dream content and emotional cascades at night (Selby et al., 2013). Cognitive and emotional processes during dreaming and wakefulness interact, and their interaction may contribute to difficulty in distinguishing whether an event/experience occurred during the waking state or in a dream.

Moreover, individuals who are more likely to make cognitive mistakes are less likely to trust their cognitive skills (van Heugten – van der Kloet et al., 2014a); thus, they may be more responsive to suggestions from other people. We hypothesize that people with BPD who have some cognitive deficits will trust their cognitive processes less because they are not sure if their perception of reality is correct. Our assumption is that due to their cognitive disturbances, persons with BPD, compared to non-clinical populations, more often will be unsure what the source of certain events or experiences are (dream vs. reality), and may even make incorrect judgments about their source. The difficulty that BPD patients encounter in reality testing (Fiqueierdo, 2006) and their problems with metacognitive monitoring and metacognitive knowledge (Sharma and Singh, 2012) seem to be the only examples of cognitive disturbances that could lead to a higher probability of experiencing DRC, compared to people from non-clinical populations.

### Thin Boundaries

The concept of boundaries, which was defined by Hartmann (2011), refers to a wide spectrum of boundaries in the mind, including interpersonal boundaries (the self vs. others), boundaries between the self and the outside world, and boundaries between different states of consciousness (e.g., the waking state or dreaming state). Thus, boundaries refer to: (i) connectedness among various aspects of the mind (i.e., the relationships among thoughts, emotions, and memory), including relationships among the personal past, future, and present; and (ii) connectedness between the self and the outside world (Hartmann, 2011). People have been characterized as having thin or thick boundaries (Hartmann et al., 1991; Hartmann, 2011). As boundaries are generally stable across situations, there is a high probability that individuals with thin boundaries in certain areas will have thin boundaries in other areas (Hartmann, 2011).

Patients with BPD tend to have thin boundaries (Hartmann, 2011), and individuals with thin boundaries have higher dream recall than those with thick boundaries. They also experience more emotions in their dreams, have dreams that are more negative and emotionally intense, dream more frequently about verbal interactions with others, and regard their dreams as more meaningful and creative (Schredl et al., 1999). A higher dream recall and other vivid night experiences are associated with a greater tendency for absorption, which is *a state of heightened imaginative involvement in which an individual’s attention capacities are focused in the behavioral domain, often to the exclusion of explicit information-processing in other domains and fantasizing* (Levin and Young, 2002, p. 203). Levin and Young (2002) demonstrated that absorption is correlated with heightened creativity, a tendency toward dissociation, and increased involvement in imagination-based activities, with concomitant alterations in consciousness (Levin and Young, 2002). In this context, it may be interesting to raise questions as to whether a greater ability to recall dreams increases the probability that dream content will be confused with real events. As BPD patients have a greater tendency for absorption (Zanarini et al., 2000), they would experience DRC more frequently, perhaps in connection with other variables discussed in this paper.

Thin boundaries seem to enhance the intrusion of dreamlike content into consciousness in the waking state, which may lead to difficulty distinguishing whether an event/experience occurred during the waking state or in a dream (Hartmann, 2011). Additionally, these intrusions may favor the occurrence of dissociative symptoms, which as mentioned above, are correlated with DRC (Rassin et al., 2001; van Heugten – van der Kloet et al., 2014a). Taken together, these data suggest that individuals with thin boundaries may be more prone to DRC. Thus, it is possible that patients diagnosed with BPD also may be more prone to DRC. Given the above considerations, it would be interesting to conduct studies on the relationship between creativity and DRC in BPD populations and non-clinical populations. Although a recent study by van Heugten – van der Kloet et al. (2015) found a moderate correlation between state dissociation and creativity, no significant correlation was found between creativity and unusual sleep experiences. Future studies should include DRC as a construct to elucidate its relationship with creativity.

## Conclusions

The variables that may lead to an increased tendency to experience DRC in patients with BPD were described in the preceding paragraphs. Taking everything into account, we propose a tentative model that patients with BPD are more prone than non-clinical population to experience difficulty or an inability to determine whether an event/experience occurred during the waking state or was part of a dream.

Patients with diagnosed BPD may be more prone to experience dissociative symptoms because of traumatic events in their early childhood (Watson et al., 2006), and dissociative symptoms are correlates of DRC (Rassin et al., 2001). Thus, the greater tendency toward dissociative symptoms among persons with BPD may make them uncertain whether something they remember happened in their dreams or while they were awake. Dissociative symptoms are correlated with fantasy proneness, which is also correlated with DRC (Rassin et al., 2001; Merckelbach et al., 2005; Giesbrecht and Merckelbach, 2006). Furthermore, persons with BPD often suffer from various sleep disorders (Hafizi, 2013), and sleep disorders (for instance, nightmares) and unusual sleep experiences are related to dissociative symptoms and to DRC (Giesbrecht and Merckelbach, 2006; van Heugten – van der Kloet et al., 2014b). Moreover, patients with BPD experience negative emotions during wakefulness that are related to negative emotional experiences during dreaming (Selby et al., 2013). This process may also be connected to elevated cognitive arousal during sleeping that causes awakenings or semi-awakenings, which consequently lead to difficulty distinguishing between dreaming and waking experiences (Trajanovic et al., 2007; Selby et al., 2013). In addition, dreams that are more likely to be confused with reality are realistic, evoke negative emotions, and give rise to behavior in the waking state (Rassin et al., 2001). The dream content of patients with BPD is more negative than the dream content of individuals in non-clinical populations, and people with BPD tend to experience a number of different cognitive disturbances, such as problems with reality testing, metacognitive deficits, altered social inference, poor decision-making skills, or cognitive distortions (Fiqueierdo, 2006; Trivedi, 2006; Wenzel et al., 2006; Schredl et al., 2012; Sharma and Singh, 2012; Mak and Lam, 2013). We hypothesize that due to the feedback loop of negative affect during dreaming and wakefulness, cognitive disturbances, and increased impulsivity, patients with BPD may take actions after awakening that are based on their dream content, because they are convinced that these events happened in reality. Moreover, people with BPD are characterized by thin boundaries (Hartmann, 2011), which may further enhance the confusion between dreams and wakefulness.

This theoretical analysis, including such variables as sleep disturbances, dissociative symptoms, negative dream content, cognitive dysfunction, and thin boundaries leads to the proposition that patients with BPD may be more prone to DRC, compared to non-clinical population. Future research on the general working model should involve the use of factor analysis and structural equation modeling in order to identify and confirm important variables and explore the complex relationships among these variables.

We plan to conduct empirical verification of these relationships. The aim of this extensive research program is not only to examine whether patients with BPD are more vulnerable than other people to experience DRC, but to study comprehensively the psychological and neuropsychological aspects of sleep and dreams, using subjective and objective methods, on a continuum: BPD – some features of BPD – lack of BPD.

The theoretical analysis presented above is exploratory in nature and is intended to serve as a starting point for further, more advanced analyses, and to provide a theoretical basis for planning empirical studies.

## Conflict of Interest Statement

The authors declare that the research was conducted in the absence of any commercial or financial relationships that could be construed as a potential conflict of interest.
